# When nausea invades your life (Psychosomatic): a case report

**DOI:** 10.1192/j.eurpsy.2023.1618

**Published:** 2023-07-19

**Authors:** E. Arroyo Sánchez, P. Setién Preciados

**Affiliations:** Psiquiatría, Hospital Universitario Príncipe de Asturias, Madrid, Spain

## Abstract

**Introduction:**

Nausea is a very common symptom related to multiple physical illnesses. In the same way, nausea and other gastrointestinal symptoms are frequently associated with the symptomatology presented at the onset of anxiety or depression. These symptoms can significantly affect the patient’s functionality, reduce school or work attendance and lead to consultation with multiple medical specialties. Therefore, it is important that in addition to a good organic screening, psychiatric pathology should be considered in the differential diagnosis. We will present the case of a 22-year-old male with nausea as the main symptom referred to Psychiatry after having seen several specialists and having undergone multiple diagnostic tests without significant findings.

**Objectives:**

To review the association of psychosomatic symptoms with anxiety disorders and/or depression, as well as their management.

**Methods:**

Presentation of a case and review of the available literature on the presence of psychosomatic symptoms, specifically nausea, in patients with anxiety and/or depression.

**Results:**

In patients in whom anxiety and depression were assessed by the Hospital Anxiety and Depression Scale (HADS), 48% reported gastrointestinal symptomatology during the previous year, of whom 12% reported nausea. It has been observed that anxiety had a higher risk for the presence of nausea (OR 3.42) than depression, although the latter also increased the risk of nausea (OR 1.47). The literature shows that interventions such as cognitive-behavioral therapy or pharmacological treatment with selective serotonin reuptake inhibitor (SSRI) drugs independently or in combination are strategies that have demonstrated therapeutic success.

**Conclusions:**

The multidimensional nature of symptomatology such as nausea and other types of psychosomatic symptoms forces us to take a broad view of the symptom. The association of gastrointestinal symptoms and pathologies such as anxiety and/or depression has been demonstrated, so that, after a correct organic screening, mental health professionals should be considered to evaluate the possible management of symptoms that can become so disabling in the life of these patients.

**Disclosure of Interest:**

None Declared

